# Radical Nephroureterectomy in the Very Elderly: A Single-Center Experience of Patients Aged ≥85 Years

**DOI:** 10.5152/tud.2026.25125

**Published:** 2026-05-13

**Authors:** Shu Gozu, Shugo Yajima, Gaku Okumura, Erika Ikezoe, Naoki Imasato, Kohei Hirose, Madoka Kataoka, Yasukazu Nakanishi, Hitoshi Masuda

**Affiliations:** Department of Urology, National Cancer Center Hospital, Chiba, Japan

**Keywords:** 85 years, complications, radical nephroureterectomy, upper tract urothelial carcinoma, very elderly

## Abstract

**Objective:**

The incidence of upper tract urothelial carcinoma is increasing with population aging, yet evidence regarding radical nephroureterectomy (RNU) in very elderly patients is limited. This study evaluated perioperative outcomes, complications, and oncological results of RNU in patients aged ≥85 years compared with younger counterparts.

**Methods:**

A total of 305 patients who underwent RNU between April 2003 and December 2024 at a tertiary institution were retrospectively reviewed. After excluding those with incomplete data (n = 4), non-urothelial histology (n = 8), or undeterminable pathology (n = 9), 284 patients were eligible. Group A included 24 patients aged ≥85 years, range 85-94 years, and group B included 260 patients <85 years, range 46-84 years. Clinicopathological features, operative outcomes, complications, and survival were compared. Temporal trends and restricted mean survival time (RMST) analyses were also performed.

**Results:**

Baseline characteristics were similar (all *P* > .05). Median laparoscopic operative time was shorter in group A (206 vs. 235 minutes, *P* = .029). Estimated blood loss, transfusion, and complication rates (10.6% overall; major 2.2%) did not differ (all *P* > .05). Pathological outcomes were comparable (all *P* > .05). Adjuvant therapy was less frequent in group A (0% vs. 21.2%, *P* = .006). Follow-up was 16.3 vs. 35.2, with no differences in survival (all *P* > .05). The RMST showed shorter survival in group A.

**Conclusion:**

The RNU appears feasible in carefully selected patients aged ≥85 years, with perioperative and oncological outcomes broadly comparable to those of younger patients. Chronological age alone should not contraindicate surgery. Restricted mean survival time offers complementary insights into survival estimation in this high-risk population.

**Highlights**

- Radical nephroureterectomy appears feasible in carefully selected patients aged ≥85 years.
- Perioperative outcomes and complication rates were comparable to those of younger patients.
- Operative time was shorter in the very elderly, likely reflecting surgical team experience and case selection.
- Long-term oncological outcomes, including recurrence-free survival, did not differ significantly by age.
- Careful patient selection and multidisciplinary perioperative management are key to favorable outcomes.

## Introduction

Upper tract urothelial carcinoma (UTUC) accounts for 5%-10% of urothelial carcinomas and often presents at an advanced stage with aggressive behavior. Radical nephroureterectomy (RNU), including excision of the bladder cuff, remains the gold standard for localized disease.^1^With global population aging, the number of elderly patients undergoing major urological procedures has increased substantially.^2^In Japan, one of the most rapidly aging societies, the proportion of citizens aged ≥85 years continues to expand, posing unique clinical challenges. Advanced age is commonly associated with diminished physiological reserves, comorbidities, and frailty, all of which may adversely impact surgical outcomes.^3^Frailty, in particular, has emerged as a critical determinant of outcomes in elderly surgical candidates. While several studies have reported perioperative and oncological outcomes in octogenarians (≥80 years), data for patients aged ≥85 years remain limited. Ishikawa et al^4^ reported inferior overall survival (OS) but comparable cancer-specific survival (CSS) in this population, suggesting competing mortality risks as the main determinant of prognosis.Similarly, Yamada et al^5^demonstrated equivalent oncological outcomes between elderly and younger patients. Recent studies have highlighted the role of minimally invasive surgery in improving perioperative safety^6^and the prognostic value of nutritional indices such as the Geriatric Nutritional Risk Index.^7^These findings underscore the importance of comprehensive assessment—including frailty, comorbidity, and nutritional status—rather than chronological age alone when determining surgical eligibility for RNU. Management decisions for UTUC should not be based on chronological age alone, but should also consider patient comorbidities, functional status, frailty, and tumor characteristics. To the best of knowledge, this study represents one of the largest single-center analyses focusing exclusively on patients aged ≥85 years undergoing RNU. By specifically targeting this very elderly population, which is rarely included in clinical trials, this study aims to provide real-world evidence regarding the perioperative feasibility and oncological outcomes of RNU. This study sought to evaluate perioperative, pathological, and survival outcomes of RNU in patients aged ≥ 85 years (group A) compared with those aged <85 years (group B). In addition, temporal trends in surgical cases were examined, and restricted mean survival time (RMST) analysis was employed to complement Kaplan–Meier estimates, aiming to provide a more clinically intuitive understanding of survival in very elderly patients.

## Material and Methods

### Patient Selection

**Figure 1:**
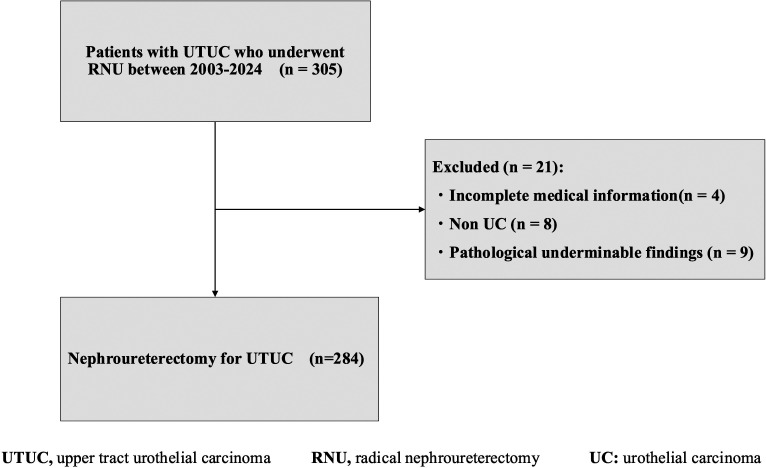
Flowchart of patient inclusion in the study. A total of 305 patients who underwent radical nephroureterectomy (RNU) for upper tract urothelial carcinoma (UTUC) between 2003 and 2024 were assessed. After excluding patients with incomplete medical information (n = 4), non-urothelial histology (n = 8), and indeterminate pathological findings (n = 9), 284 patients were included in the analysis.

Between April 2003 and December 2024, 305 patients underwent RNU at the institution. As shown in Figure 1, patients with incomplete medical records (n = 4), non-urothelial carcinoma histology (n = 8), or undeterminable pathological findings (n = 9) were excluded, leaving 284 eligible patients. Patients were divided into 2 groups: group A (aged ≥85 years, range 85-94 years) and group B (aged <85 years, range 46-84 years). The age cutoff of 85 years was selected to define a very elderly population in Japan’s super-aging society, as patients in this age group are rarely included in surgical outcome studies or clinical trials.

### Data Collection

Baseline variables included age, sex, body mass index (BMI), Eastern Cooperative Oncology Group performance status (ECOG PS), Charlson Comorbidity Index (CCI), tumor location, history of bladder cancer, hydronephrosis, and clinical stage. Operative variables included surgical approach (open, laparoscopic, robot-assisted), operative time, estimated blood loss (EBL), and need for perioperative transfusion. Estimated blood loss was defined as intraoperative blood loss recorded by the anesthesiology team. Pathological features included tumor stage, nodal status, lymphovascular invasion, concomitant carcinoma in situ (CIS), and margin status. Details of perioperative systemic therapy, including chemotherapy regimens, are summarized in Table 1, 2. Notably, no patients aged ≥85 years received adjuvant chemotherapy.

### Surgical Techniques

Surgical approaches comprised open, laparoscopic, and robot-assisted procedures. Open surgery predominated until 2012. Thereafter, laparoscopic and subsequently robot-assisted RNU were increasingly utilized, with the da Vinci Xi® system (Intuitive Surgical, Sunnyvale, CA, USA) introduced in 2022. Surgical approach was determined by surgeon preference, patient condition, and available resources. The choice of surgical modality was not randomized but determined by surgeon preference, patient comorbidities and performance status, and the temporal availability of minimally invasive platforms. In general, minimally invasive approaches were preferred in elderly and frail patients when feasible. The distribution of surgical approaches in each group is summarized in Table 2.

### Complications

Postoperative complications within 90 days were graded according to the Clavien–Dindo classification. Major complications were defined as grade ≥ III. Detailed complication categories were documented, including gastrointestinal, infectious, cardiovascular, and wound-related events. The 90-day time frame was selected to capture both early and delayed surgery-related complications.

### Statistical Analysis

Normality of continuous variables was assessed using the Shapiro–Wilk test, and the Mann–Whitney *U*-test was applied when the data did not follow a normal distribution. Categorical variables were analyzed using the *χ*^2^ test or Fisher’s exact test, as appropriate. To account for potential confounders, multivariable Cox proportional hazards analyses were performed. The model included selected covariates: age group, CCI, ECOG performance status, surgical approach, and adjuvant therapy, as well as established pathological prognostic factors. Survival outcomes were estimated with the Kaplan–Meier method and compared using the log-rank test. Restricted mean survival time was also calculated to complement conventional survival analysis. All statistical analyses were performed using R software version 4.3.1, with *P *< .05 considered statistically significant.

### Ethical Considerations

This retrospective study, which utilized an opt-out methodology, was exempt from the requirement for individual informed consent. All patients, however, provided written informed consent for their surgical treatment. The study protocol was approved by the institutional ethics committee of National Cancer Center Hospital East (approval number 2018-159; date 2018/8/6).

## Results

### Baseline Characteristics

Median age was 74 years (range 46-94 years). Group A had a median age of 86 (85-94 years). No significant differences in sex distribution, BMI, ECOG PS, comorbidity burden, tumor laterality, or history of bladder cancer were observed. Median follow-up was significantly shorter in group A (16.3 vs. 35.2 months, *P*= .002) (Table 1).

### Operative Outcomes

Surgical approach distribution was similar across groups: open (43.7%), laparoscopic (34.5%), and robot-assisted (21.8%). Operative times for open and robotic approaches did not differ, but laparoscopic procedures were shorter in group A (median 206 vs. 235 min, *P *= .029). Estimated blood loss and transfusion requirements were comparable (Table 2).

### Pathological Outcomes

Pathological stage distribution was similar, with pT1 41%, pT2 16%, pT3 41%, and pT4 3%. Nodal status, lymphovascular invasion, and concomitant CIS did not differ. Positive margins were rare (4.6% overall) and absent in group A. Adjuvant therapy was administered in 21.2% of group B but in none of group A (*P *= .006) (Table 2).

### Complications

Overall, 30 patients (10.6%) experienced postoperative complications, with 6 (2.2%) classified as major. Specific complications included ileus (2.5%), urinary tract infection (1.8%), chylous leakage (0.7%), bleeding (1.4%), wound issues (1.1%), cardiovascular events (1.4%), and pulmonary complications (0.4%). No significant differences were observed between group A and group B (Table 3).

### Patient Trends and Survival Outcomes

**Figure 2:**
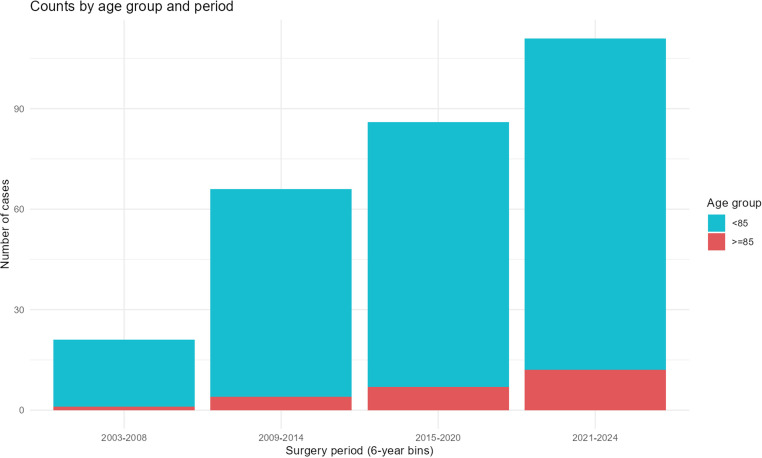
Number of patients aged ≥ 85 years undergoing RNU stratified by age group. Number of patients aged ≥85 years (group A) and <85 years (group B) undergoing radical nephroureterectomy across 4 consecutive time periods (2003–2008, 2009–2014, 2015–2020, and 2021–2024). Although the absolute number of very elderly patients increased, their proportion remained stable over time.

**Figure 3:**
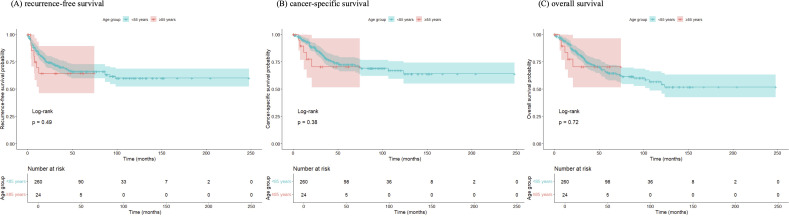
Kaplan–Meier curves for (A) recurrence-free survival, (B) cancer-specific survival, and (C) overall survival. Kaplan–Meier curves demonstrate (A) recurrence-free survival (RFS), (B) cancer-specific survival (CSS), and (C) overall survival (OS) in patients aged ≥85 years (group A) and <85 years (group B). No statistically significant differences were observed between groups (RFS: *P* = .49; CSS: *P* = .38; OS: *P* = .72).

**Figure 4:**
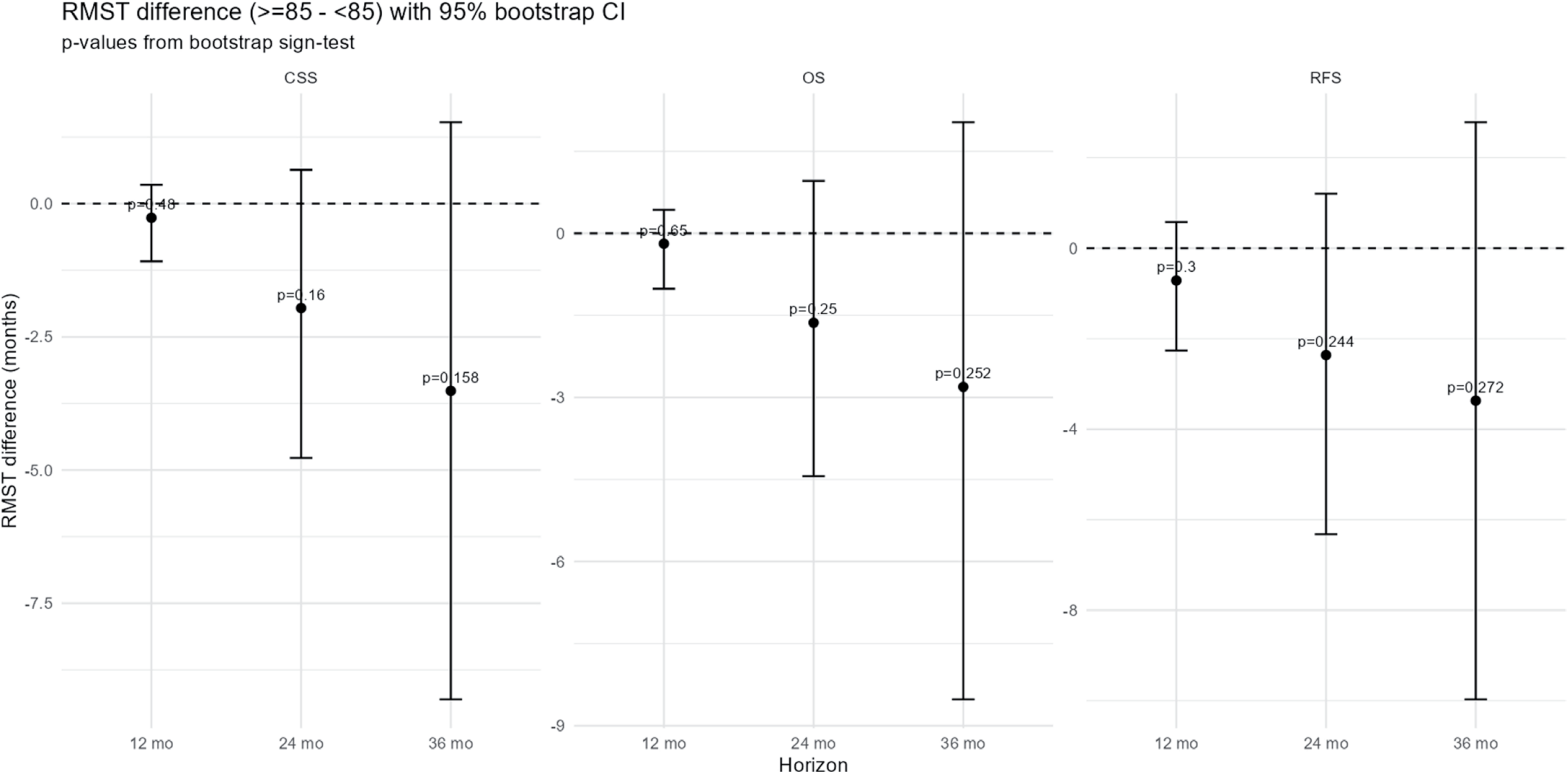
Restricted mean survival time (RMST) difference (≥85 to <85) with 95% bootstrap CI. Differences in RMST (≥85-<85 years) at 12, 24, and 36 months are shown for cancer-specific survival (CSS), overall survival (OS), and recurrence-free survival (RFS), with 95% bootstrap CIs and *P* values obtained from bootstrap sign tests. Negative values indicate shorter RMST in patients aged ≥85 years.

**Figure 5:**
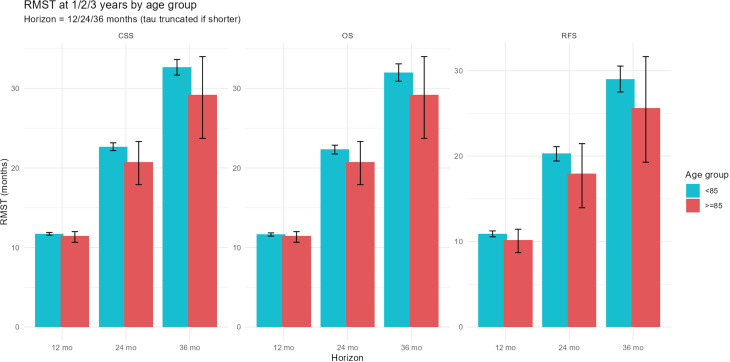
RMST at 1, 2, and 3 years by age group. RMST estimates at 12, 24, and 36 months for CSS, OS, and RFS are displayed for group A (≥85 years) and group B (<85 years). Bars represent mean RMST with 95% CIs. No significant differences were observed between groups.

**Figure 6:**
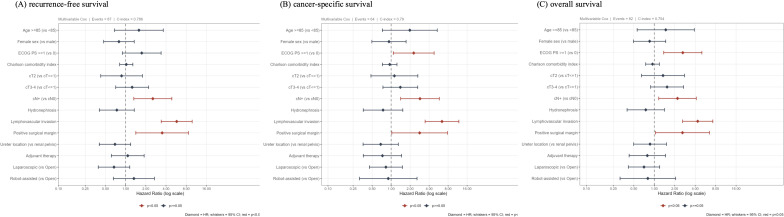
Multivariable Cox proportional hazards regression analyses for recurrence-free survival (A), cancer-specific survival (B), and overall survival (C). Forest plots display hazard ratios (HRs) with 95% CIs for each covariate included in the multivariable models. The analyses adjusted for selected clinical and pathological factors, including age group (≥85 vs. <85 years), Charlson comorbidity index, ECOG performance status, surgical approach, and adjuvant therapy.

Recurrence-free survival (RFS), CSS, and OS were analyzed. The number of patients aged ≥85 years undergoing RNU increased over time in parallel with overall surgical cases, though their proportion remained stable (Figure 2). During follow-up, recurrence occurred in 7 patients (29.2%) in group A and 80 patients (30.7%) in group B. Cancer-specific deaths were observed in 5 (20.8%) and 59 (22.7%), respectively. The 1-year RFS was 69.6% vs. 83.3%, and the 3-year RFS was 64.3% vs. 70.7%, with similar results for CSS and OS (RFS, *P *= .492; CSS, *P *= .383; OS, *P *= .717). Kaplan–Meier curves showed overlapping trajectories (Figure 3), indicating preserved short-term outcomes in the very elderly. The RMST analysis showed numerically shorter survival in group A, with a mean difference of approximately –2.5 months at 2 years (95% CI –7 to +2) for OS, –2.5 months (95% CI –5 to 0) for CSS, and –3.0 months (95% CI –8 to +2) for RFS. These differences did not reach statistical significance. The shorter follow-up in group A (16.3 vs. 35.2 months) may also have led to underestimation of late events (Figures 4 and 5). In multivariable Cox regression analysis (Figure 6), age ≥85 years was not independently associated with survival outcomes.

## Discussion

In this context, the present study represents one of the largest single-center cohorts focusing exclusively on patients aged ≥85 years undergoing RNU and provides real-world perioperative and oncological outcome data in a population that has been largely underrepresented in previous surgical outcome studies. This study demonstrated that RNU appears feasible in carefully selected patients aged ≥85 years, with perioperative and oncological outcomes largely comparable to younger patients. However, these findings should be interpreted cautiously given the small sample size and potential selection bias. Importantly, operative time, blood loss, and postoperative complications were not adversely influenced by age alone. The findings align with recent reports. Ishikawa et al^4^reported equivalent CSS across age groups but inferior OS in ≥85-year-old patients, primarily due to competing mortality. Similarly, Yamada et al^5^found comparable oncological outcomes between elderly and younger patients. It is acknowledged that lymph node dissection was not uniformly performed, resulting in a substantial proportion of pNx cases. This reflects real-world practice, particularly in very elderly or frail patients, and may have led to understaging. Ye et al^8^ showed that advanced age should not be an absolute contraindication to RNU, and Wang et al^9^reported survival benefits from radical surgery in elderly UTUC patients compared to conservative management. In this cohort, the absolute number of patients aged ≥85 years undergoing RNU increased over time, reflecting demographic aging and the necessity to treat symptomatic cases (e.g., gross hematuria, hydronephrosis, infection). This emphasizes that decisions were driven not by age itself but by clinical necessity. The RMST analysis provided complementary insights, offering an alternative perspective to conventional Kaplan–Meier estimates in this very elderly cohort. While survival in patients aged ≥85 years appeared numerically shorter, these differences did not reach statistical significance. Therefore, RMST results should be interpreted as exploratory and primarily hypothesis-generating rather than conclusive. Nevertheless, this approach may enhance the clinical interpretability of outcomes in elderly populations where follow-up duration is limited and competing risks are prevalent. The heterogeneity of surgical approaches represents an important limitation of this study. Surgical modality was determined by surgeon preference, patient condition, and temporal availability of minimally invasive platforms rather than randomization. Therefore, the present study does not aim to establish comparative superiority between age groups, but rather to describe real-world perioperative feasibility and oncological outcomes of RNU in very elderly patients. Beyond chronological age, recent evidence has highlighted the importance of geriatric screening tools in predicting surgical outcomes and tailoring perioperative management in elderly patients with urological cancers. Yajima and Masuda emphasized the clinical value of the G8 and other geriatric assessments as practical instruments to capture frailty and vulnerability, thereby facilitating individualized treatment decisions. Incorporating such tools may complement conventional oncological evaluation and help refine patient selection for RNU in the very elderly.^10^ Shorter laparoscopic operative times in group A may reflect selection bias favoring minimally invasive approaches in frail patients, as well as accumulated institutional expertise. Prior studies reported that minimally invasive RNU offers reduced blood loss, faster recovery, and equivalent cancer control in elderly patients. A critical challenge remains the absence of adjuvant therapy in very elderly patients.^11^ This imbalance in adjuvant therapy may have influenced survival outcomes and should be considered when interpreting the results. This is likely attributable to postoperative renal function decline after nephroureterectomy, frailty, and comorbidity burden, which frequently preclude eligibility for cisplatin-based chemotherapy. In this context, optimal surgical management may play an even more critical role in disease control for very elderly patients who are unfit for systemic therapy. Leow et al^12^noted that while use of perioperative chemotherapy is increasing, its feasibility is limited in frail individuals. Comprehensive geriatric assessment, nutritional optimization, and multidisciplinary collaboration may broaden access to multimodal therapy. Follow-up duration was significantly shorter in the ≥85-year group, which may have led to underestimation of late recurrence and cancer-specific mortality, reflecting frailty and competing risks. However, inadequate surveillance may delay detection of recurrence. Novel approaches such as telemedicine, home-based monitoring, and caregiver involvement could improve continuity of care.

### Clinical Implications

Radical nephroureterectomy should not be withheld solely on the basis of age in carefully selected patients aged ≥85 years. Individualized assessment incorporating frailty, comorbidities, and symptomatic burden is crucial. Minimally invasive surgery and geriatric-focused perioperative care may enhance safety and quality of life.

### Limitations

First, patients aged ≥85 years in this cohort likely represent a highly selected population with relatively preserved performance status and manageable comorbidity burden. In addition, the number of very elderly patients who were evaluated but not offered surgery could not be reliably determined from the medical records. Second, the small sample size of the ≥85-year group limits statistical power, particularly for survival analyses. Standardized geriatric assessment tools such as the G8 screening test, frailty index, sarcopenia assessment, and ADL status were not consistently available in this retrospective cohort. This study is limited by its retrospective, single-institutional design and small sample size in Group A, which may reduce statistical power. Shorter follow-up may underestimate late recurrence or cancer-related mortality. Unmeasured confounders such as socioeconomic status and patient preference may also have influenced outcomes. Furthermore, the interval between UTUC diagnosis and subsequent development of bladder cancer has been suggested as a prognostic indicator. However, this factor could not be uniformly assessed in the cohort due to inconsistent documentation inherent to the retrospective design. Future prospective studies capturing detailed longitudinal data will be necessary to clarify its oncological impact. The relatively short follow-up period reflects the recent increase in very elderly patients and their limited life expectancy. Physical and cognitive limitations leading to difficulties in outpatient follow-up and a high rate of non-cancer-related mortality also contributed.

### Future Perspectives

Future multicenter prospective studies incorporating frailty indices, nutritional assessments, and patient-reported outcomes are warranted. Robotic platforms and Enhanced Recovery After Surgery protocols tailored to the elderly may further improve safety. Integration of telemedicine and community healthcare resources may enhance follow-up and survivorship care in very elderly patients.

## Conclusion

Radical nephroureterectomy appears feasible in carefully selected patients aged ≥85 years (group A), achieving perioperative and oncological outcomes comparable to group B. Chronological age alone should not contraindicate surgery; instead, individualized assessment of performance status and comorbidity burden is paramount. However, challenges including limited access to adjuvant therapy and difficulty in sustaining long-term follow-up remain. Larger prospective multicenter studies and tailored supportive care strategies are essential to refine management and improve outcomes for this growing population.

## Figures and Tables

**Table 1.** Clinical Characteristics of Patients in Group A and Group B

**Variable**	**Total** **(n = 284)**	**Group A** **(n = 24)**	**Group B** **(n = 260)**	*P*
Age (years), median (range)	74 (46-94)	86 (85-94)	73 (46-84)	<.001
Follow-up (months), median (range)	33.9 (0.3-247.6)	16.3 (0.3-74.5)	35.2 (0.4-247.6)	.002
Sex, n (%) Male Female	205 (72.2) 79 (27.8)	14 (58.3) 10 (41.7)	191 (73.5) 69 (26.5)	.151
BMI (kg/m^2^), median (range)	22.6 (11.2-31.5)	22.3 (16.4-26.6)	22.6 (11.2-31.5)	.585
ECOG PS, n (%) 0 1	260 (91.5) 24 (8.5)	20 (83.3) 4 (16.7)	240 (92.3) 20 (7.7)	.131
CCI, n (%) 0 1 2 3 6	208 (73.2) 61 (21.4) 13 (4.6) 1 (0.4) 1 (0.4)	15 (62.5) 8 (33.3) 1 (4.2) 0 0	193 (74.2) 53 (20.4) 12 (4.6) 1 (0.4) 1 (0.4)	.403
Laterality, n (%) Right Left	144 (50.7) 140 (49.3)	14 (58.3) 10 (41.7)	130 (50.0) 130 (50.0)	.524
History of bladder cancer, n (%) Absent Present	251 (88.4) 33 (11.6)	21 (87.5) 3 (12.5)	230 (88.5) 30 (11.5)	.749
Hydronephrosis, n (%) Absent Present	132 (46.5) 152 (53.5)	12 (50.0) 12 (50.0)	120 (46.2) 140 (53.8)	.831
Tumor location, n (%) Renal pelvis Ureter Both	140 (49.3) 143 (50.3) 1 (0.4)	15 (62.5) 9 (37.5) 0	125 (48.1) 134 (51.5) 1 (0.4)	.272
cT stage, n (%) ≤ 1 2 3 4	116 (40.8) 65 (22.9) 99 (34.9) 4 (1.4)	7 (29.2) 5 (20.8) 11 (45.8) 1 (4.2)	109 (41.9) 60 (23.1) 88 (33.8) 3 (1.2)	.247
cN stage, n (%) 0 1 2	259 (91.2) 13 (4.6) 12 (4.2)	23 (95.8) 0 1 (4.2)	236 (90.8) 13 (5.0) 11 (4.2)	.716
Neoadjuvant therapy, n (%) Absent Present	268 (94.4) 16 (5.6)	24 (100.0) 0	244 (93.8) 16 (6.2)	.377
Neoadjuvant therapy regimen, n (%) GC Gcarbo	9 (56.3) 7 (43.7)	0 0	9 (56.3) 7 (43.7)	–
Preoperative creatinine (mg/dL), median (range)	0.9 (0.5-2.4)	0.9 (0.6-2.4)	0.9 (0.5–2.2)	.639

BMI, body mass index; CCI, Charlson Comorbidity Index; ECOG PS, Eastern Cooperative Oncology Group performance status; GC, Gemcitabine+Cisplatin; Gcarbo, Gemcitabine + Carboplatin.

Group A, patients aged ≥ 85 years; Group B, patients aged <85 years.

**Table 2.** Operative and Pathological Characteristics of Group A and Group B

**Variable**	**Total** **(n = 284)**	**Group A** **(n = 24)**	**Group B** **(n = 260)**	*P*
Operative method, n (%) Open Laparoscopic Robot-assisted	124 (43.7) 98 (34.5) 62 (21.8)	10 (41.7) 9 (37.5) 5 (20.8)	114 (43.9) 89 (34.2) 57 (21.9)	.962
Operative time (min), median (range) Open Laparoscopic Robot-assisted	243.5 (78–566) 234 (40–426) 199.3 (130–278)	226.5 (78–392) 206 (40–244) 196.8 (130–240)	244.5 (129–566) 235 (116–426) 199.5 (135–278)	.419 .029 .868
EBL (mL), median (range) Open Laparoscopic Robot-assisted	219.5 (0–2100) 44.0 (4–3376) 21.5 (3–716)	192.5 (17–778) 39.0 (10–267) 75.0 (13–258)	219.5 (0–2100) 44.0 (4–3376) 21.0 (3–716)	.588 .740 .183
Perioperative blood transfusion, n (%) Open Laparoscopic Robot-assisted	10 (8.1) 3 (3.1) 1 (1.6)	1 (10.0) 0 0	9 (7.9) 3 (3.4) 1 (1.8)	.583 1.000 1.000
Pathological T stage, n (%) ≤ pT1 pT2 pT3 pT4	116 (40.8) 45 (15.8) 115 (40.5) 8 (2.8)	12 (50.0) 4 (16.7) 8 (33.3) 0	104 (40.0) 41 (15.8) 107 (41.1) 8 (3.1)	.772
Pathological N stage, n (%) pN0 pN+ pNx	76 (26.7) 26 (9.2) 182 (64.1)	2 (8.3) 2 (8.3) 20 (83.4)	74 (28.5) 24 (9.2) 162 (62.3)	.067
Concomitant CIS, n (%) Absent Present	208 (73.2) 76 (26.8)	20 (83.3) 4 (16.7)	188 (72.3) 72 (27.7)	.336
LVI, n (%) Absent Present	174 (61.3) 110 (38.7)	14 (58.3) 10 (41.7)	160 (61.5) 100 (38.5)	.828
Positive surgical margin, n (%) Negative Positive	271 (95.4) 13 (4.6)	24 (100.0) 0	247 (95.0) 13 (5.0)	.611
Adjuvant therapy, n (%) Not received Received	229 (80.6) 55 (19.4)	24 (100.0) 0	205 (78.8) 55 (21.2)	.006
Adjuvant therapy regimen, n (%) GC Gcarbo Nivolumab	41 (74.5) 9 (16.4) 5 (9.1)	0 0 0	41 (74.5) 9 (16.4) 5 (9.1)	–

CIS, Carcinoma in situ; EBL, Estimated blood loss; GC, Gemcitabine+Cisplatin; Gcarbo, Gemcitabine+Carboplatin; LVI, Lymphovascular invasion.

Group A, patients aged ≥ 85 years; Group B, patients aged <85 years.

**Table 3.** Complication Category and Clavien–Dindo Classification of Group A and Group B

**Category or Classification**	**Total** **(n = 284)**	**Group A** **(n = 24)**	**Group B** **(n = 260)**	*P*
Complication category, n (%) Ileus Urinary tract infection Chylous leakage Postoperative bleeding Wound infection Wound dehiscence Cardiovascular events Urinary retention Anastomotic leakage Cholecystitis Pulmonary fistula	7 (2.5) 5 (1.8) 2 (0.7) 4 (1.4) 1 (0.4) 2 (0.7) 4 (1.4) 4 (1.4) 2 (0.7) 1 (0.4) 1 (0.4)	0 2 (8.3) 0 0 0 0 0 0 0 0 0	7 (2.7) 3 (1.2) 2 (0.8) 4 (1.5) 1 (0.4) 2 (0.8) 4 (1.5) 4 (1.5) 2 (0.8) 1 (0.4) 1 (0.4)	–
Clavien–Dindo classification, n (%) I II IIIa IIIb Total	3 (1.1) 21 (7.4) 3 (1.1) 3 (1.1) 30 (10.6)	0 2 (8.3) 0 0 2 (8.3)	3 (1.2) 19 (7.3) 3 (1.2) 3 (1.2) 28 (10.8)	0.867

Group A, patients aged ≥ 85 years; Group B, patients aged <85 years.

## Data Availability

The data that support the findings of this study are available on request from the corresponding author.
